# Endoscopic evacuation of supratentorial hematoma: A hemostatic strategy for surgeons

**DOI:** 10.1097/MD.0000000000036501

**Published:** 2025-01-17

**Authors:** Tiancai Lan, Shoutang Liu, Yuanliang Ye, Jiale Zhu, Ruixiang Wei, Chuanming Wang, Guirong Ma

**Affiliations:** a Department of Neurosurgery, Liuzhou People’s Hospital, Liuzhou, Guangxi Autonomous Region, China; b Engineering Technological Research Center for Nervous Anatomy and Related Clinical Applications, Liuzhou, Guangxi Autonomous Region, China, ^c^ Department of Neurology, Huazhong University of Science and Technology Union Shenzhen Hospital, Shenzhen, Guangdong province, China.

**Keywords:** endoscopic surgery, hemostatic strategy mortality, intracerebral hemorrhage

## Abstract

Endoscopic hematoma evacuation has become well received for its high evacuation rate in patients with intracerebral hematoma. Effective hemostatic procedure is the key to the success of the procedure. Any single method cannot solve all kinds of intraoperative bleeding, The key to hemostasis is to identify the type of bleeding and take the best hemostasis method during endoscopic surgery. In our study, sixty-two intracerebral hemorrhage patients who underwent endoscopic hematoma evacuations were analyzed. Intraoperative bleeding was graded as Grades 0, 1, 2, and 3 based on characteristics of bleeding. A hemostatic strategy was created from the grading system. The efficiency was evaluated by operation time, evacuation rate, and re-bleeding rate after surgery. Procedure safety was evaluated by mortality rate and postoperative complications. We found that endoscopic removal of putamen hematoma was more prone to intraoperative bleeding (*P* = .00). Active bleeding occurred in early operative stage and errhysis happen in later stage (*P* = .00). Average evacuation rate was 95.61% and the mortality rate was 3.23%. The mean Glasgow outcome scale (GOS) score at 6-month follow-up was 3.77 ± 1.12. No patient experienced postoperative re-bleeding. These findings indicated that most patients will experience different degrees of intraoperative bleeding during endoscopic hematoma evacuation. A hemostatic strategy based on intraoperative bleeding grade resulted in efficiency and safety.

## 1. Introduction

A spontaneous intracerebral hematoma (ICH) is one of the most deadly forms of stroke with extremely high mortality rate.^[1–5]^ Only 20% of patients can survive in 6 months and the cost of survival is a great economic burden to everyone.^[6,7]^ Endoscopic hematoma evacuation is an effective and safe minimal invasive technique for ICH.^[8–11]^ Compare with craniotomy, endoscopic hematoma evacuation obtain higher evacuation rate and lower complications.^[11–13]^

In an operation, a transparent plastic sheath is inserted into the hematoma, and the hematoma is gradually evacuated by manipulating the suction through the working space within the sheath.^[14,15]^ However, even a careful atraumatic evacuation can result in intraoperative hemorrhage. Steep learning curve, active bleeding and limited surgical exposure are limitations of this procedure, especially for junior surgeons.^[14]^ The incidence rate of postoperative re-bleeding can be 0% to 20%.^[15–18]^

In order to prevent intraoperative bleeding, the hard blood clot adhering to hematoma cavity should not be removed with force, but leaving partial hard blood clot remain in brain may reduce the hematoma evacuation rate. Some hemostatic methods, including irrigation,^[19]^ hemostatic matrix application,^[17]^ compression with cotton,^[15]^ and coagulation with bipolar^[20–22]^ or monopolar,^[23]^ were used to deal with intraoperative bleeding during endoscopic or microscopic hematoma evacuation. However, any single method cannot solve all kinds of intraoperative bleeding. Some bleeding may hide in normal brain tissue and can be coagulated by bipolar or monopolar, making it difficult for removal simultaneously. Moreover, unreasonable hemostatic methods can result in iatrogenic normal brain tissue injury, postoperative brain edema, and even re-bleeding.

In this retrospective study, we analyzed the characteristics of intraoperative bleeding and established a hemostatic strategy based on intraoperative bleeding grade during endoscopic hematoma evacuation. We also evaluate the effectiveness and safety of this hemostatic strategy.

## 2. Materials and methods

### 2.1. Patients

**Figure 1.**
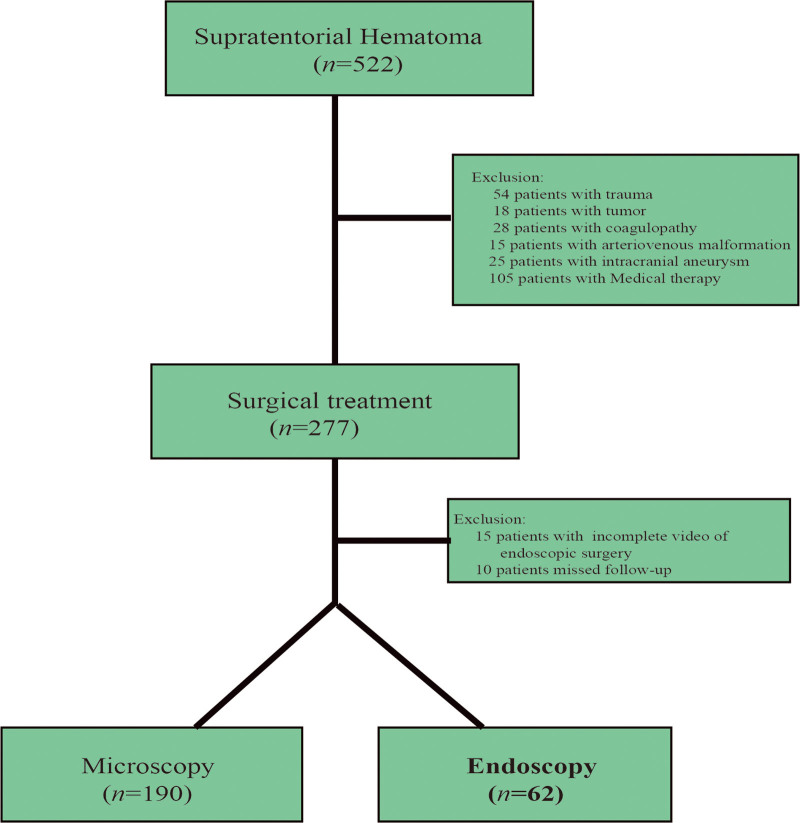
STROBE flowchart of Supratentorial Hematoma.

This study included 62 patients with supratentorial intracerebral hematoma (ICH) between January 2015 and July 2022 at the Neurosurgery Department of the Liuzhou People Hospital affiliated to Guangxi Medical University (Fig. 1). All patients underwent endoscopic hematoma evacuation with or without decompressive craniectomy. To observe the characteristics of intraoperative bleeding, the entire operative video of each patient was reviewed in detail. The study was approved by the ethics committee of the Liuzhou people Hospital. The need for individual consent was waived by the committee because of the retrospective nature of the study.

### 2.2. Inclusion and exclusion criteria

Patient selection for endoscopic evacuation of hematomas: a putamen ICH with a hematoma volume >30 mL; a subcortical hemorrhage >30 mL with significant mass effect (midline shift >5 mm and compression of mesencephalic cistern) and neurological deterioration; a thalamic ICH with a hematoma volume >20 mL and intraventricular hemorrhage (IVH) with acute hydrocephalus; Hematoma removal after ruptured aneurysm embolization. Patient deselection for endoscopic evacuation of hematomas: ICH caused by trauma, tumor, coagulopathy, or arteriovenous malformation; patients taking antiplatelet or anticoagulation medications; hematomas was scattered, involving multiple cerebral cortex; Exclusion criteria: patients who did not have operative video and without a follow-up CT scan within 3 days after surgery; the patient family or legal representative didn’t consent to surgical treatment.

### 2.3. Surgical procedures

For putamen ICH, we used transfrontal or transtemporal approach for hematoma evacuation. For patients with cortical hemorrhage, we used transcortical approach through the corridor that traverses the shortest distance to the center of hematoma (determined by preoperative CT scan). In patients with a thalamic ICH and IVH, we used the ipsilateral Kocher point as our entry point with free-hand technique to evacuate the blood. The surgical procedure was performed with the patient in the supine or lateral position. A single curved 4-cm skin incision was made on the entry site. A small craniotomy was widened to 20 to 25 mm in diameter, dura mater was incised and opened, a small sized pial incision on the cortex was performed, and then the transparent plastic sheath was inserted to the core of the hematoma (the distance based on the preoperative CT scan). The rigid endoscope measuring 0 degrees and 4 mm in diameter (Karl Storz, German) was held in left hand, while a suction cannula was held in right hand. While the assistant holding the sheath, the rigid endoscope and suction cannula were introduced into the hematoma cavity through the endoscopic corridor. The most distal part of the hematoma was evacuated first via suction. As the sheath was gradually withdrawn the residual hematoma was removed. Sometimes the hematoma was hard and was removed by forceps. When an intraoperative bleeding occurs, we adopted our hemostatic strategy based on the intraoperative bleeding grading system below. With meticulous hemostasis, a drainage tube was not routinely set into the hematoma cavity. An ICP monitor may be inserted as needed.

### 2.4. Relationship between culprit vessels and hematoma

**Figure 2.**
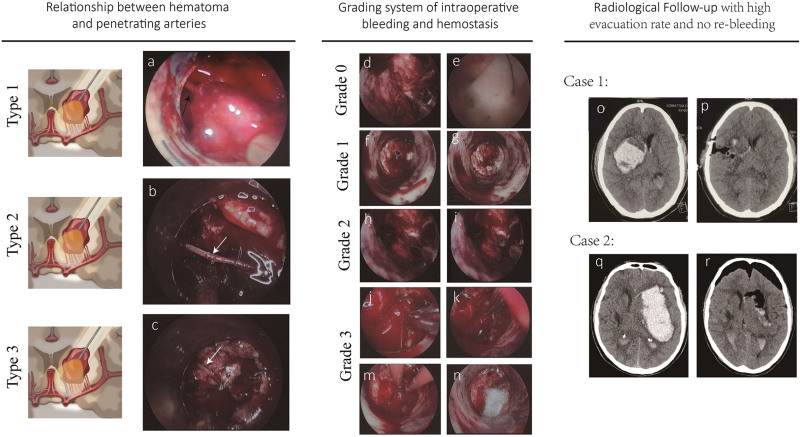
Schematic diagram illustrating the intraoperative culprit vessels, grading system of intraoperative bleeding and clinical outcome. The bleeding vessels could be inside (Type 1, a) or passing through (Type 2,b) or at the edge of the hematoma (Type 3,c). Grading system based on bleeding speed and vessels: grade 0, no bleeding with collagen sponge only (D, E); grade 1, active bleeding with the hemostatic material (F, G); grade 2, active bleeding site located in distal segment of vessel, hemostasis with irrigation-suction coagulation technique (H, I); grade 3, active bleeding site located in junction between brain tissue and vessels, hemostasis with monopolar coagulation, bipolar and pressure hemostatic material at last (J–M). Clinical cases with no re-bleeding after surgery (N–Q).

We distinguished surrounding penetrating arteries from the bleeding vessels that caused hematoma based on their anatomic characteristics. The bleeding vessels could be inside (Fig. 2: Type 1,a) or passed through the hematoma (Fig. 2: Type 2,b), supplied normal brain tissues. Other kind of normal vessels were located at the edge of hematoma (Fig. 2: Type 3,c) and adhere tightly.

### 2.5. Grading system of intraoperative bleeding and hemostasis

Table 1 presents the hemostatic strategy based on intraoperative bleeding grade intraoperative bleeding grading system used in this study. This grading system is based on bleeding speed and vessels. Grade 0 means no bleeding, water in transparent plastic sheath was clear and the scale of the sheath wall was clearly visible; Grade 1 means Errhysis, the water in the sheath was cloudy, bleeding vessels were not seen but the scale of the sheath wall was seen vaguely. Grade 2 indicates active bleeding, bleeding vessels were seen and bleeding site was located in distal segment of the vessel. Grade 3 also indicates active bleeding, bleeding site was located in junction between brain tissue and vessels. The hemostatic strategy was based on this grading system. Apply only collagen sponge or nothing at all for grade 0 bleeding (Fig. 2D and E); Locate and compress bleeding area for a few minutes with a hemostatic material (Absorbable oxidized regenerated cellulose Hemostat; Johnson & Johnson, Inc., Somerville, USA), and with repeated irrigation for grade 1 bleeding (Fig. 2F and G); Apply repeated irrigation-suction coagulation technique to found bleeding vessels for grade 2 bleeding (Fig. 2H and I). Hemostasis with monopolar and bipolar (after bleeding reduction) in turn and press hemostatic material at the end to found bleeding area and with repeated irrigation for grade 3 bleeding (Fig. 2J and M).

**Table 1:** Grading system of intraoperative bleeding and hemostasis during endoscopic hematoma evacuation.

Grade	Characteristics of intraoperative bleeding	Hemostatic strategy	Type of culprit vessels
0	No bleeding, water and mark(cotton) in transparent plastic sheath was clear.	only collagen sponge or nothing at all.	Not found
1	Errhysis, the water and mark (cotton) in the sheath was cloudy and vague, bleeding vessels can’t be seen.	Compress bleeding area for a few minutes with a hemostatic material and with repeated irrigation.	Not found
2	Active bleeding, bleeding vessels were seen and bleeding site was located in distal segment of vessels.	Irrigation-suction coagulation technique.	The bleeding vessels could be inside (Type 1) or passed through the hematoma (Type 2).
3	Active bleeding, bleeding site was located in junction between brain tissue and vessels.	Hemostasis with monopolar and bipolar in turn and press hemostatic material with repeated irrigation.	The bleeding vessels were located at the edge of hematoma and adhere tightly (Type 3).

### 2.6. Clinical and radiological follow-up

All patients underwent a follow-up CT scan within 3 days after surgery (Fig. 2N–Q). Endoscopic operation time, numbers of hemostasis in different grade, stage of appearance in different grade during operation-time, morbidity and mortality were recorded. Hematoma volume was measured by Slicer software and hematoma evacuation rate calculated as follows: [preoperative hematoma volume - postoperative hematoma volume]/preoperative hematoma volume × 100%. The 6-month mortality and long-term modified Rankin Scale score (mRS) were collected from patients’ medical records on re-admission, from the outpatient clinic records, or via telephone interviews.

### 2.7. Statistical analysis

All statistical analyses were performed using *SPSS* 22 for Windows (*SPSS* Inc., Chicago, Illinois). Continuous variables were expressed as mean ± standard deviation. Categorical variables were expressed in frequency or as a percentage. Pearson Chisquare tests were used to determine any statistical difference about proportions. Continuous variables were compared using independent t-test or Mann–Whitney *U* test. A *P* value of less than .05 was considered statistical significant.

## 3. Results

### 3.1. Baseline characteristics

There were 62 patients enrolled in the study, including 42 cases of putamen hemorrhage, 9 cases of thalamic hemorrhage, 5 cases of intraventricular hemorrhage and 6 cases of lobar cerebral. Males and females represented 70.97% and 29.32 % of patients, respectively. The mean age of the population was 53.32 ± 11.67 years. All patients underwent surgery within 24 hours of ictus. Forty-six patients underwent pure endoscopic hematoma evacuation, 16 patients were treated with hematoma evacuation and decompressive craniectomy (Table 2).

**Table 2:** General data and clinical outcome of the 62 patients with spontaneous supratentorial ICH.

Variable	Value
Age (yr)*	53.32 ± 11.67
Gender	
Male	44 (70.96%)
Female	18 (29.32%)
Location	
Putamen	42 (67.74%)
Thalamus	9 (14.52%)
Ventricle	5 (8.06%)
Lobar	6 (9.68%)
ICH volume (mL)*	
Pre	53.68 ± 18.90
Pro	3.19 ± 4.35
Operation time (min)*	
Endoscopic time	39.94 ± 17.69
Craniotomy time	42.26 ± 20.45
Blood loss*	50.23 ± 19.51
Medical morbidity	
Hypertension	45 (72.58%)
Renal insufficiency	3 (4.83%)
Diabetes mellitus;	10 (16.12%)
Entry point	
Transfrontal	45 (72.58%)
Transtemporal	11 (17.74%)
Transcortical	6 (9.68%)
Mortality rate (%)	3.23
Postoperative rebleeding rate (%)*	0
Pre-GCS	7.45 ± 2.24
Pro-GCS (6 month)	12.71 ± 2.83
Pro-GOS (6 month)	3.77 ± 1.12
Stage of bleeding appearance(%)*,†	
Grade 1	68.68 ± 20.95
Grade 2	47.16 ± 24.80
Grade 3	31.24 ± 17.56

GCS = Glasgow coma scale; GOS = Glasgow outcome scale, ICH = intracerebral hematoma.

* Values are expressed as the mean ± SD.

† Grade 3 bleeding occurred in early operative stage, with statistical significance (*P* = .000).

### 3.2. Intraoperative bleeding

The numbers of hemostasis with patient in grade 1, 2 and 3 were 55, 18 and 24 respectively (*P* = .000) (Table 3). Compared with thalamic hematoma and lobar hematoma removal, endoscopic putamen hematoma removal was more prone to intraoperative bleeding (*P* = .038) (Table 3). When the endoscopic operating time was divided into one hundred equal parts, the average stage of bleeding appearance per patient in grade1, 2 and 3 were 68.68 ± 20.95, 47.16 ± 24.80 and 31.24 ± 17.56 respectively, these mean active bleeding occurred in early operative stage and errhysis happened later (*P* = .000) (Table 2).

**Table 3:** Characteristics of intraoperative bleeding with endoscopic hematoma evacuation.

Grade	Operative stage			Location of hematoma		
	Early- stage	Mid- stage	Later-stage	Putamen	Thalamus	Lobar
1	2 (2.06)	11 (11.34)	42 (43.30)	37 (38.14)	10 (10.31)	8 (8.25)
2	5 (5.15)	10 (10.30)	3 (3.09)	30 (30.93)	1 (1.03)	2 (2.06)
3	19 (19.59)	4 (4.12)	1 (1.03)	22 (22.68)	1 (1.03)	1 (1.03)
	χ^2^ = 66.022 *P* = .000			χ^2^ = 10.17 *P* = .038		

### 3.3. Clinical outcome

**Figure 3.**
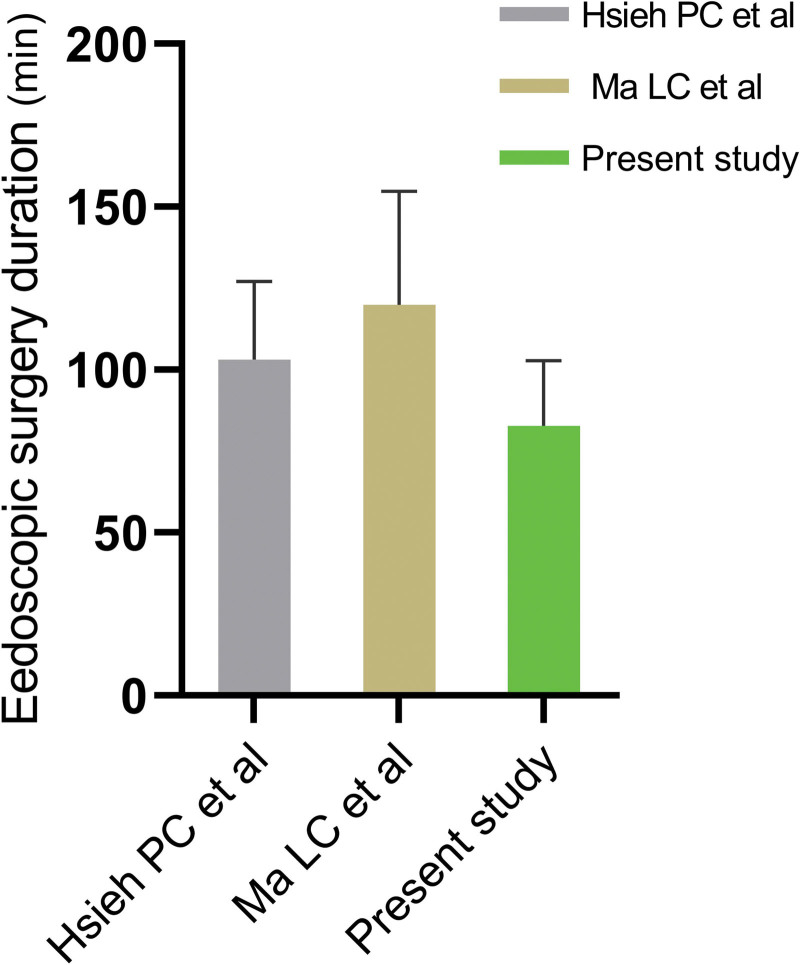
Comparing with Hsieh PC et al^[2]^ and Ma LC et al,^[14]^ Endoscopic surgery for intracerebral hemorrhage with grading system of intraoperative bleeding and hemostasis required shorter operation time.

The mean preoperative hematoma volume was 53.68 ± 18.90 mL and the mean residual hematoma volume was 3.19 ± 4.35 mL, representing an average evacuation rate of 95.61%. Endoscopic operating time was 39.94 ± 17.69 minutes (Fig. 3) and blood loss was 50.56 ± 20.19 mL. No patient experienced postoperative re-bleeding. The mortality rate was 3.23% (2 of 62 patients), 1 died of renal failure, one from pneumonia and sepsis. Surgery-related morbidity wasn’t occurred in all patients. The mean preoperative Glasgow coma scale (GCS) score was 7.45 ± 2.24 and the mean GCS score 6 months after surgery was 12.71 ± 2.83. The mean GOS score at 6-month follow-up was 3.77 ± 1.12 (Table 2).

## 4. Discussion

Endoscopic surgery has been gaining popularity as a method for surgical intervention in ICH patients. Although the International Surgical Trial (STICH)in intracerebral hemorrhage study showed that emergency surgical hematoma evacuation failed to improve the outcome of ICH patient comparing with the same type of patients who received initial medical management without surgery, the STICH trial further clarify demonstrated improved outcomes with surgery for subgroups. A meta-analysis of 15 trials of surgery for ICH including STICH I and II with individual patient data showed a significant advantage for surgery. Compare with craniotomy, endoscopic hematoma evacuation obtain some advantage:higher evacuation rate and lower complications; exposure of deep structure and hemostasis under direct vision; maximum protection of normal brain tissue.

Intraoperative bleeding during endoscopic hematoma removal is a stressful factor for surgeons. Even careful atraumatic evacuation of a hematoma can sometimes result in intraoperative hemorrhage. The principals for surgical hemostasis with intraparenchymal hematoma with endoscopic vs microscopic technique were not different. Also, whether surgery is performed through a tube (endoscopic visualization) or retractors (microscope for visualization) does not change principals of hemostasis. Some methods can be used to acquire secure hemostasis in endoscopic hematoma evacuation (Table 4). When an intraoperative hemorrhage occurs, the operating field was kept clear by irrigation and continuous suction. Hemorrhage from a small artery could be reduced with repeated irrigation in hematoma cavity. Another way was to compress the bleeding spot with cotton or hemostatic material for several minutes. Hemorrhage from a large artery can be stopped by coagulation with suction cannula or bipolar.^[10,15–17,19]^

**Table 4:** Summary of the literature on hemostatic procedures for endoscopic evacuation of intracerebral hemorrhage.

Authors and years	Surgical hemostatic procedures	No. of pts	Outcome
Chad M Miller, et al (2007)	The hematoma cavity was irrigated with saline and hemostasis was inspected via endoscope.	10	The re-bleeding rate was 20% and the mortality rate was 25%.
Toru Nagasaka, et al (2009)	Handling of the multifunctional suction cannula and its application for balanced irrigation-suction.	15	No surgical complications or re-bleeding occurred.
Lu-Ting Kuo, et al (2011)	Most bleeding from these perforators stopped after gentle compression with cotton and irrigation for 2 minutes.	68	The re-bleeding rate was 1.5% and the mortality rate was 5.9%.
Hongwei Zhu, et al (2012)	Hemostasis was achieved by continuous saline irrigation, pressure packing and electrically coagulated with a suction cannula.	28	The re-bleeding rate was 3.6% and the mortality rate was 7.1%.
Wei-Hsin Wang, et al (2015)	Hemostasis was achieved by hemostatic agents or used bipolar forceps with the endoscope held by an assistant or holder.	21	The re-bleeding rate was 9.52% and the mortality rate was 9.52%.
Hui-Tzung Luh, et al (2018)	FloSeal Hemostatic Matrix was applied through a specialized 3-mm flexible catheter into the hematoma cavity.	42	The re-bleeding rate was 4.8% and the mortality rate was 7.1%.
Present study	Hemostatic strategy for endoscopic evacuation of intracerebral hematoma based on intraoperative bleeding grading system.	62	No patient experienced postoperative re-bleeding and the mortality rate was 3.23%.

As we know, single or multiple branches of the penetrating arteries are responsible for cerebral bleeding. The causes of hemorrhages include arteriosclerosis, arterial dissection and micro-aneurysm formation, etc.^[24–26]^ The lenticulostriate arteries (LSA) represent one of the largest groups of perforating vessels in the brain and divided into 2 groups (medial and later) based on the originative location of the vessels.^[27]^ The lenticulostriate arteries originate from the middle cerebral artery (MCA), and then turn sharply medial, make a loop, and penetrate anterior perforated substance into the brain.^[28]^ Intracerebral segments of the LSA supply the Putamen, lateral segment of Globus Pallidus and internal capsule.^[29]^ The diameter of lenticulostriate arteries vary from 0.7um to 2.2mm.^[30]^ The intraoperative hemorrhage is often iatrogenic induce by injury of perforating vessels inside or outside the hematoma. Based on the anatomic characteristics of penetrating arteries, the relationship between the hematoma/brain tissue and the penetrating arteries can been speculated. For the intracerebral hematoma, the bleeding vessels can run inside or passed through the hematoma, supplying normal brain tissues. Other kind of normal vessels are located at the edge of hematoma and adhere tightly.

When encountering intraoperative hemorrhage, the best choice of hemostatic method can be bewildered especially for inexperienced surgeons. It was invalid in active hemorrhage with the methods of observation and compression. Errhysis with coagulation would transfer to active hemorrhage because of brain tissue damage around small arteries. When active hemorrhage occurs, the operating field is filled with blood quickly, bipolar was difficult to use because of narrow corridor and visual control deteriorates. Based on intraventricular vision, some researches presented grading system and a step-by-step guide for the endoscopic management of intraoperative hemorrhages during intraventricular tumor surgery.^[31]^ Based on the surgical sites, anatomical structure and bleeding vessels between the intraventricular surgery and intracerebral hematoma, we established a simple intraoperative bleeding grading system during endoscopic hematoma remove. This grading system can guide inexperienced surgeon to choose optimum hemostatic method and is safe and effective.

The following points warrant attention with intraoperative bleeding grading system and hemostasis. First, to distinguish no bleeding, errhysis and active bleeding based on the characteristics of the water in transparent plastic sheath. No bleeding means clear water. Errhysis means light-red cloudy water and fuzzy reference (cotton or scale on the sheath). Active bleeding included red and muddy water, even malignant hemorrhage. Second, to find bleeding site or vessels through repeated irrigation and continuous suction, to coagulate the bleeder by touching the suction with appropriative power of monopolar. Third, to reduce the damage of normal brain tissue in Grade3 by using the outer wall of suction (not the tip) to touch the bleeding site.

In our study, the concept of hematoma removal was to avoid following the brain parenchyma–hematoma junction during hematoma evacuation, therefore, the incidence of Grade3 was less than that of Grade1 and Grade2. The perforator vessels were located in the bottom of the hematoma and the lower part of the hematoma was removed first, so that the period in Grade3 was earlier than Grade1.

## 5. Limitations

The major strength of this study was to provide an alternative hemostatic strategy to neurosurgeons. A hemostatic strategy based on intraoperative bleeding grade resulted in efficiency and safety. The present study has some limitations. Firstly, evaluation of bleeding in endoscopy was largely subjective and likely to have interobserver variations. Secondly, the hemostatic strategy was dependent upon the intraoperative bleeding grading system, which could introduce a bias. In addition, more clinical experience was necessary to determine whether this strategy improves surgical outcomes.

## 6. Conclusion

Active bleeding occurs in early operative stage and errhysis happens in late stage. Hemostatic strategy based on intraoperative bleeding grading system can guide surgeon to choose the right hemostatic methods, shorten the operation time, and seems to be safe and effective, especially for inexperienced surgeons.

## Author contributions

**Conceptualization:** Yuanliang Ye, Shoutang Liu, Chuanming Wang, Guirong Ma.

**Data curation:** Yuanliang Ye, Tiancai Lan, Jiale Zhu, Ruixiang Wei, Guirong Ma.

**Methodology:** Tiancai Lan, Jiale Zhu, Ruixiang Wei.

**Resources:** Yuanliang Ye.

**Supervision:** Tiancai Lan, Shoutang Liu, Chuanming Wang, Guirong Ma.

**Writing – review & editing:** Shoutang Liu, Guirong Ma.
